# Our Friends Must Write

**Published:** 1887-12

**Authors:** 


### OUR FRIENDS MUST WRITE.

We thank all who have written for us in the past. We hope they will do so again, and we urge others to write. If you have anything practical or interesting, whether it be the report of a case in practice, a useful formula, or anything which you have reason to believe will benefit the profession, send it to us, and let it go upon record. Sometimes a note requesting information of medical brethren on some case or point in practice will elicit important suggestions from brethren in the profession. It is thus that we may hope to develop the medical literature and promote the progress of medical science in the world. Let us not be laggards in the profession.

